# Nociception monitors vs. standard practice for titration of opioid administration in general anesthesia: A meta-analysis of randomized controlled trials

**DOI:** 10.3389/fmed.2022.963185

**Published:** 2022-08-25

**Authors:** Dandan Ma, Jiahui Ma, Huayong Chen, Dongliang Mu, Hao Kong, Lingzhi Yu

**Affiliations:** ^1^Department of Pain Management, Jinan Central Hospital, Shandong University, Jinan, China; ^2^Department of Anesthesiology, Yidu Central Hospital Affiliated to Weifang Medical University, Weifang, China; ^3^Department of Anesthesiology, Peking University First Hospital, Beijing, China

**Keywords:** general anesthesia, nociception, analgesia nociception index, nociception monitors, opioid

## Abstract

**Background:**

Nociception monitors are being increasingly used during surgery, but their effectiveness in guiding intraoperative opioid administration is still uncertain. This meta-analysis of randomized controlled trials (RCTs) aimed to compare the effectiveness of nociception monitors vs. standard practice for opioid administration titration during general anesthesia.

**Methods:**

We searched the electronic databases of PubMed, EMBASE, Cochrane Library, Clinical Trial, and Web of Science from inception up to August 1, 2021, to identify relevant articles, and extracted the relevant data. Intraoperative opioid administration, extubation time, postoperative pain score, postoperative opioid consumption and postoperative nausea and vomiting (PONV) were compared between patients receiving nociception monitoring guidance and patients receiving standard management. The standardized mean difference (SMD), with 95% confidence interval (CI), was used to assess the significance of differences. The risk ratio (RR), with 95% CI, was used to assess the difference in incidence of PONV. Heterogeneity among the included trials was evaluated by the *I*^2^ test. RevMan 5.3 software was used for statistical analysis.

**Results:**

A total of 21 RCTs (with 1957 patients) were included in the meta-analysis. Intraoperative opioid administration was significantly lower in patients receiving nociception monitor-guided analgesia than in patients receiving standard management (SMD, −0.71; 95% CI, −1.07 to −0.36; *P* < 0.001). However, pain scores and postoperative opioid consumption were not significantly higher in the former group. Considerable heterogeneity was found among the studies (92%). Extubation time was significantly shorter (SMD, −0.22; 95% CI, −0.41 to −0.03; *P* = 0.02) and the incidence of PONV significantly lower (RR, 0.78; 95% CI, 0.61 to 1.00; *P* = 0.05) in patients receiving nociception monitoring guidance.

**Conclusions:**

Intraoperative nociception monitoring guidance may reduce intraoperative opioid administration and appears to be a viable strategy for intraoperative titration of opioids.

**Systematic review registration:**

https://www.crd.york.ac.uk/prospero/display_record.php?RecordID=273619, identifier: CRD42019129776.

## Introduction

Recent studies suggest that nociception monitors reflect perception of injury under anesthesia more accurately than standard practice (which is based on changes in vital signs) (1) and, therefore, should be used to guide intraoperative analgesia. Use of nociception monitors has been shown to reduce risk of opioid overtreatment, opioid induced hyperalgesia and adverse reactions, and to shorten wake-up time after general anesthesia (2–6). Several studies have demonstrated that nociception monitors have greater sensitivity to a variety of clinical stimuli and even allow prediction of patient body movements in response to nociceptive stimuli (7, 8). However, previous studies have not always been consistent (9, 10). Jiao et al. found that nociception monitors during general anesthesia reduced the use of intraoperative opioids (11), but their meta-analysis included only three kinds of nociception monitors; moreover, only a few studies examined the efficacy of analgesia nociception index (ANI) and pupillary pain index (PPI), and so their conclusions may be biased.

In recent years, there have been many new studies on nociception monitors, and several new nociception monitors have been introduced. There is a need to reevaluate the value of nociception monitors in light of the fresh evidence. This meta-analysis of randomized controlled trials (RCTs) was performed to evaluate the effect of nociception monitors vs. standard practice on intraoperative opioid administration—the primary outcome—and extubation time, postoperative pain, postoperative opioid consumption, and incidence of postoperative nausea and vomiting (PONV)—the secondary outcomes—in patients undergoing surgery under general anesthesia. The findings of this meta-analysis will provide anesthetists with a rational strategy for intraoperative opioid titration.

## Methods

The study was conducted according to the Preferred Reporting Items for Systematic Reviews and Meta-Analyses (PRISMA) guidelines (12). It was registered in the international prospective register of systematic reviews with ID No. CRD42019129776 (https://www.crd.york.ac.uk/prospero/display_record.php?RecordID=273619). The PRISMA checklist is available in Supplementary Table 1.

### Search strategy

Two investigators independently searched five electronic databases—PubMed, EMBASE, Cochrane Library, Clinical Trial, and Web of Science—to identify relevant articles published from inception of the database to August 1, 2021. The search was conducted using the following Medical Subject Headings terms and corresponding keywords: (“analgesia nociception index” OR “ANI” OR “skin conductance” OR “pupillometry” OR “nociceptive flexion reflex threshold” OR “NFR threshold” OR “SPI” OR “surgical stress Index” OR “qNOX” OR “IoC_2_” OR “nociception level index” OR “NoL” OR “surgical pleth index” OR “SSI”) AND (“Nociception” OR “Monitoring, Physiologic”) AND (“Anesthesia, General”). The search strategy used in PubMed is described in detail in Supplementary Table 2. If the full texts of the articles could not be accessed, the original information was requested from the authors. The reference lists of the selected articles were searched to identify additional relevant studies.

### Inclusion and exclusion criteria

Articles were eligible for inclusion in this meta-analysis if (1) the study was an RCT; (2) the study population included patients of all ages undergoing surgery under general anesthesia; (3) opioid administration was compared between patients receiving nociception monitors and standard practice; (4) numerical data were provided on opioid administration, extubation time, pain score, postoperative opioid consumption and the incidence of PONV.

Animal or cadaveric studies; unpublished data or repeated data; non-randomized trials; and studies in languages other than English were excluded.

### Data extraction

Two authors independently extracted data from the selected studies. Disagreements were resolved by discussion until consensus was reached or by consulting a third author. The following data were extracted: (1) name of first author, (2) year of publication, (3) journal name, (4) country, (5) type of surgery, (6) age range, (7) sample size, (8) monitoring the depth of anesthesia, (9) anesthesia method, (10) types of nociception monitors, (11) type of opioid, (12) purpose of the research, (13) intraoperative opioid administration, (14) extubation time, (15) postoperative opioid consumption, (16) postoperative pain score, and (17) postoperative adverse events.

The extracted data were entered into a predefined standardized Excel (Microsoft Corporation, USA) file. For continuous data, we calculated the mean and standard deviation (SD). If not provided, we used de Luo's method (13) to calculate the mean and SD from the median and interquartile range.

### Assessment of risk of bias

Two authors independently evaluated the risk of bias according to the methods described in the Cochrane Handbook for Systematic Reviews of Interventions (14); any inconsistencies were resolved by discussion with the senior author. Seven items were evaluated: random sequence generation (selection bias), allocation concealment (selection bias), blinding of participants and personnel (performance bias), blinding of outcome assessment (detection bias), incomplete outcome data (attrition bias), selective reporting (reporting bias), and other bias. For each type of bias, the risk was graded as “high”, “uncertain”, or “low”.

### Statistical analysis

The standardized mean difference (SMD), with the 95% confidence interval (CI), was used to assess differences between groups in continuous data (i.e., opioid administration, extubation time, pain score, and postoperative opioid consumption). The risk ratio (RR), with the 95% CI, was used to assess differences in dichotomous data (i.e., the incidence of PONV). The results from all of the studies (either SMD or RR) were pooled using a random effect model to take into account the clinical and methodologic heterogeneity between studies. Statistical heterogeneity was analyzed using the chi-square test and *I*^2^ test. An *I*^2^ values higher than 50% was indicated moderate-to-high heterogeneity. For Subgroup analysis was conducted according to the type of nociception measurement, classification of surgery (15), age, intraoperative opioid, and anesthetic agent used. Additionally, considering that neuromuscular block reversal can shorten the extubation time, an additional subgroup analysis was made when pooling the extubation time. Publication bias was evaluated by funnel plot if the meta-analysis included more than ten studies.

Sensitivity analysis was performed by removing individual studies and recalculating the SMD to identify the studies responsible for the heterogeneity. All statistical analysis was performed using RevMan 5.3 (The Cochrane Collaboration, Oxford, England).

## Results

### Study identification and characteristics

**Figure 1:**
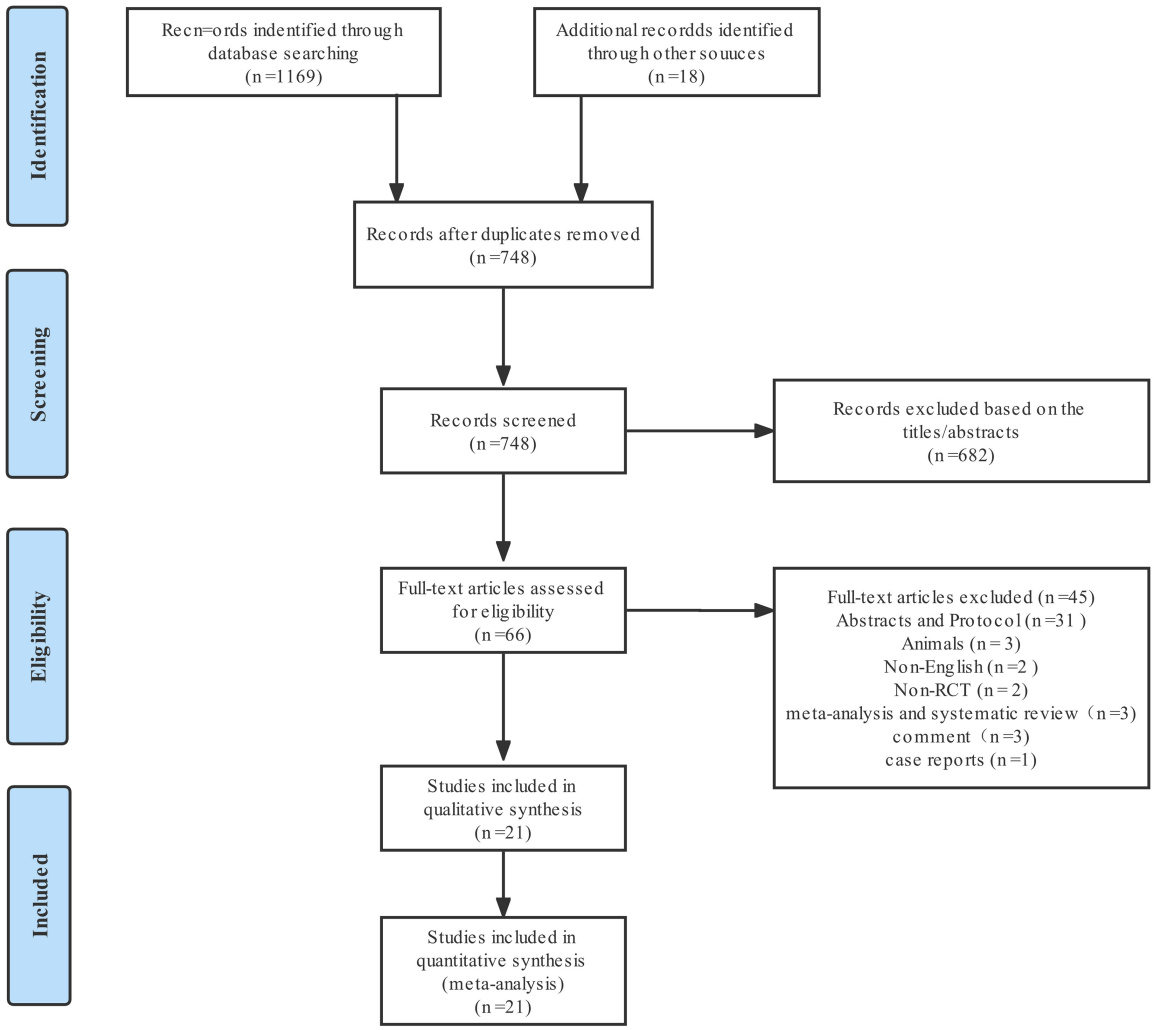
Flowchart showing the selection of studies.

From the 1,169 articles initially identified, 21 studies (2–4, 6, 9, 10, 16–29), with a total of 1,957 patients, met the eligibility criteria and were included in present meta-analysis. Figure 1 shows the study selection process; Supplementary Table 3 summarizes the characteristics of the 21 RCTs. The effects of five nociception monitors—surgical pleth index (SPI), ANI, PPI, nociception level (NoL), and indexes of consciousness (IoC_2_)—on intraoperative opioid administration were compared with that of standard practice. SPI was used for 1,058 patients, PPI for 318 patients, ANI for 273 patients, NoL for 201 patients, and IoC_2_ for 107 patients.

### Risk of bias

**Figure 2:**
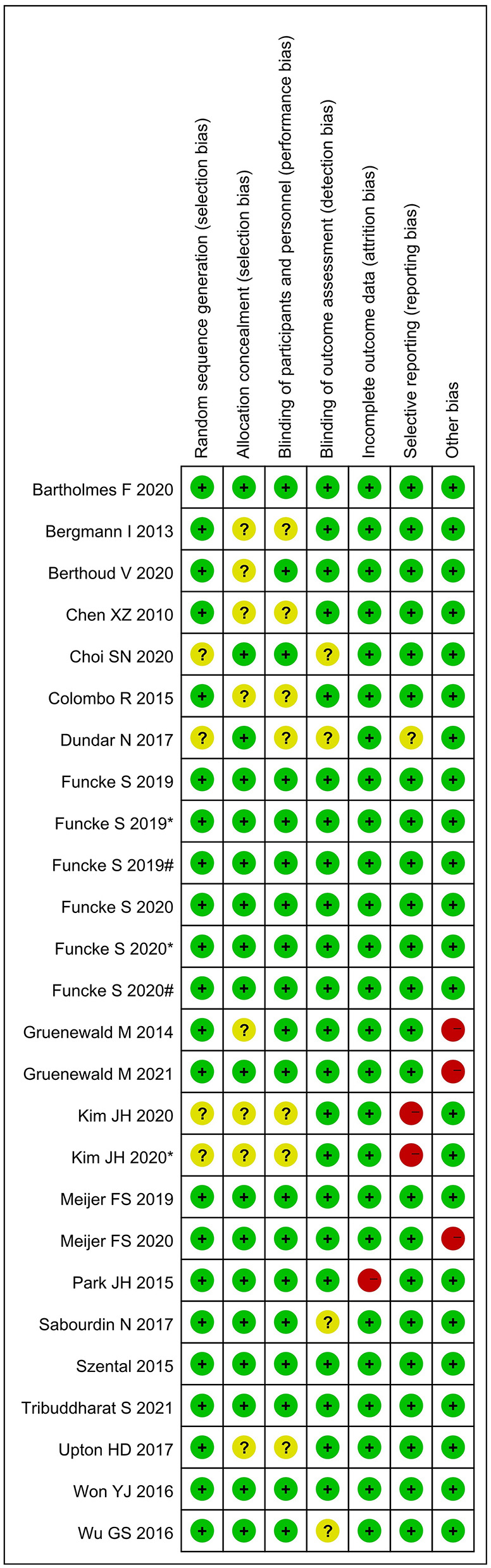
Assessment of risk of bias. “+” = low risk of bias; “?” = unclear risk of bias; and “–” = high risk of bias; # and * represent different interventions in the same study.

Figure 2 shows the assessment of risk of bias. Random sequence generation was found in eighteen studies; however, seven studies did not reveal the allocation methods. Six studies did not definitively describe blinding of the study performers, and four studies did not definitively describe blinding of the outcome assessors. One study did not present complete results data, two were selective reports, and three had other types of bias. All studies were assessed as having low risk of bias.

### Primary outcome

**Figure 3:**
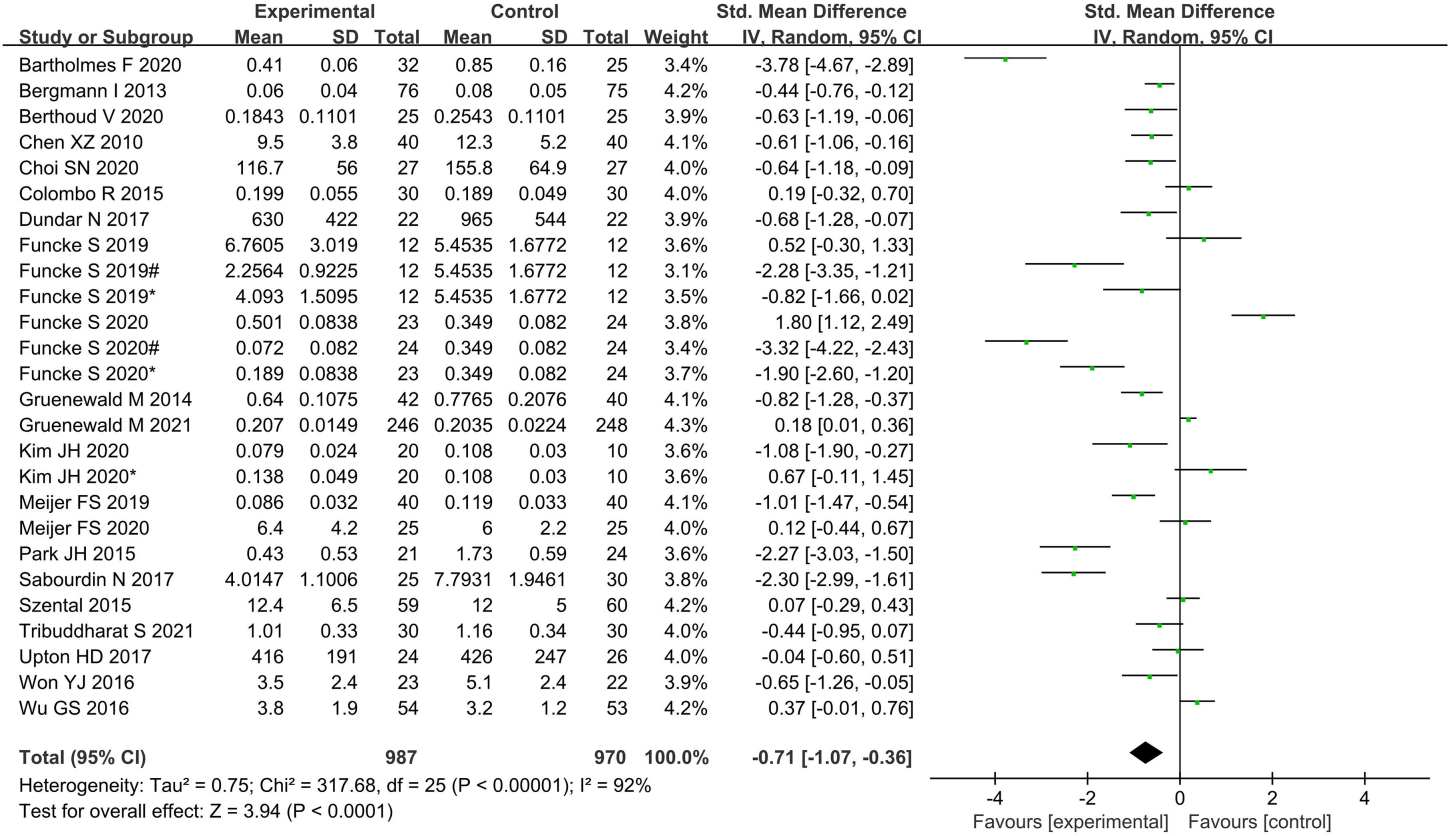
Forest plot showing the effects of intervention (nociception monitoring guidance/standard practice) on intraoperative opioid administration.

The random-effects model was applied since there was high heterogeneity (*I*^2^ = 92%; *P* < 0.001). Meta-analysis suggested that intraoperative opioid administration was significantly lower in the nociception monitor-guided group than in the standard-practice group (SMD, −0.71; 95% CI, −1.07 to −0.36). Figure 3 presents the detailed results.

### Secondary outcomes

**Figure 4:**
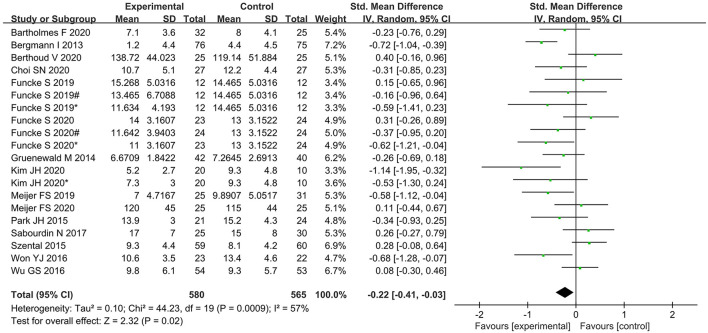
Forest plot showing the effects of intervention (nociception monitoring guidance/standard practice) on extubation time.

Time to extubation was significantly shorter in the nociception monitor-guided group than in the standard-practice group (SMD, −0.22; 95% CI, −0.41 to −0.03. The heterogeneity was relatively moderate (*I*^2^ = 57%; *P* = 0.02; Figure 4).

**Figure 5:**
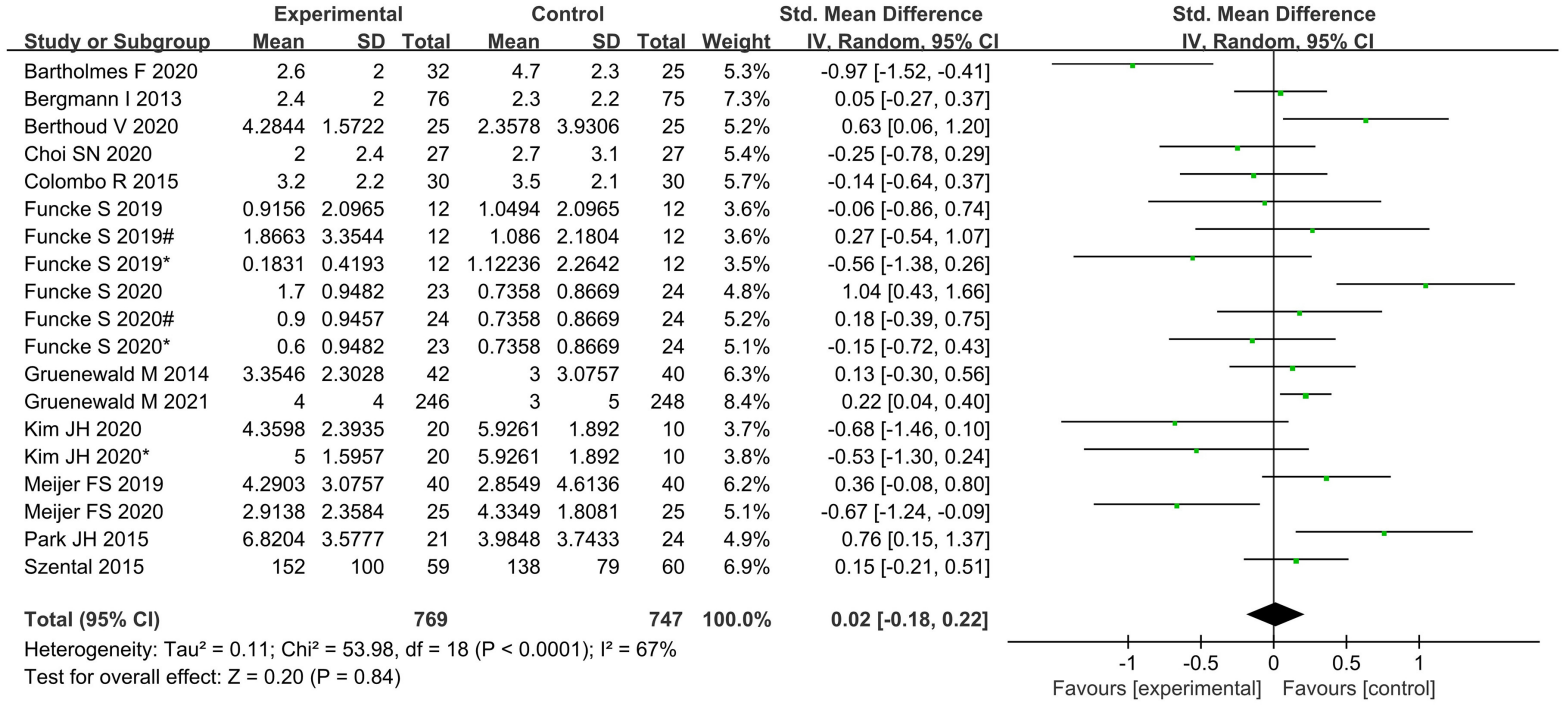
Forest plot showing the effects of intervention (nociception monitoring guidance/standard practice) on postoperative pain scores.

**Figure 6:**
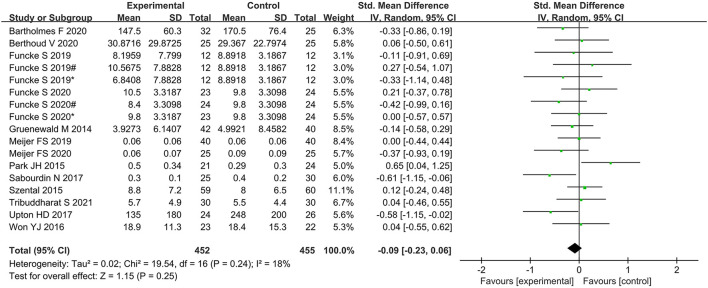
Forest plot showing the effects of intervention (nociception monitoring guidance/standard practice) on postoperative opioid consumption.

No significant difference was found between the nociception monitor-guided group and the standard-practice group in postoperative pain score (SMD, 0.02; 95% CI, −0.18 to 0.22; *I*^2^ = 67%; *P* = 0.84) and postoperative opioid consumption (SMD, 0.09; 95% CI, −0.23 to 0.06; *I*^2^ = 18%; *P* = 0.25; Figures 5, 6).

**Figure 7:**
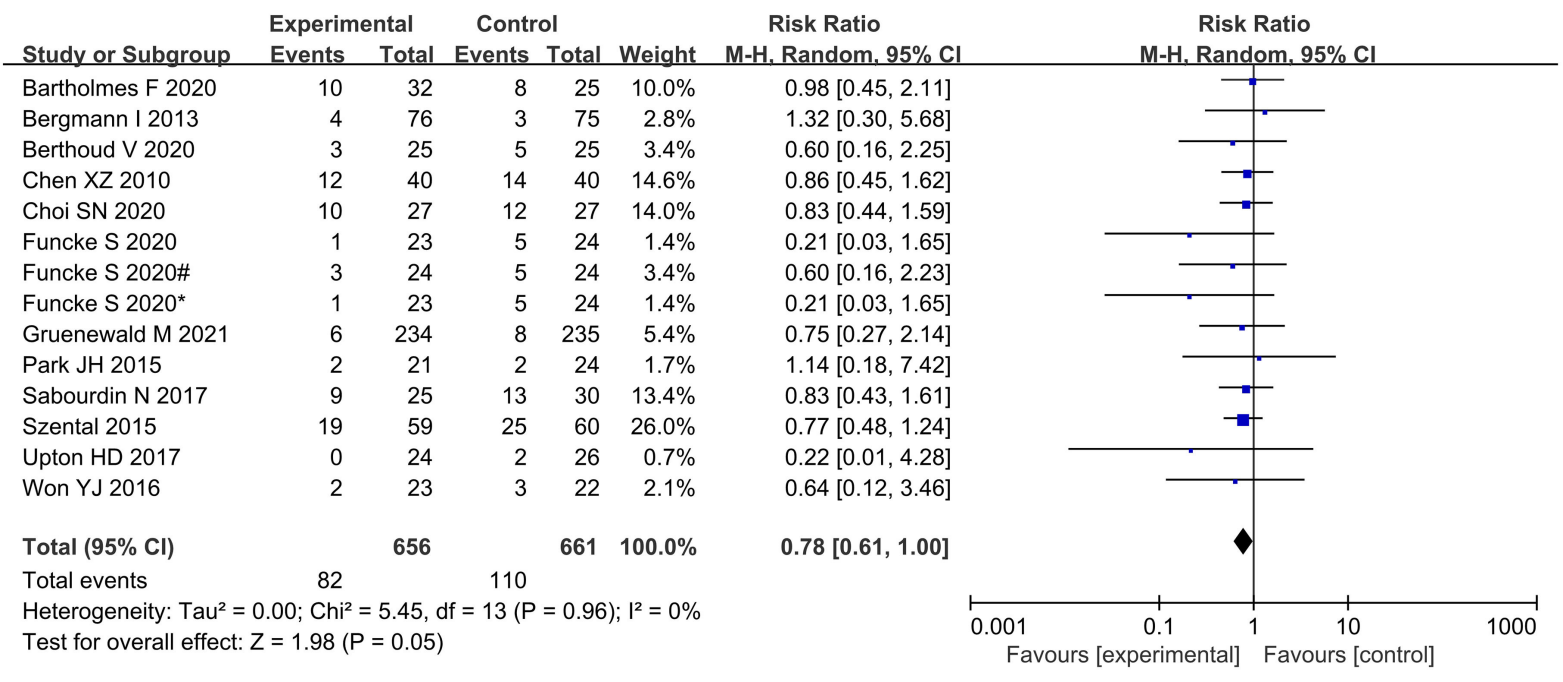
Forest plot showing the effects of intervention (nociception monitoring guidance/standard practice) on the incidence PONV.

The incidence of PONV was significantly lower in the nociception monitor-guided group than in the standard-practice group (pooled RR, 0.78; 95% CI, 0.61 to 1.00; *I*^2^ = 0%; *P* = 0.05; Figure 7).

### Subgroup analysis

As the overall results of the primary and secondary outcomes were heterogeneous (*I*^2^ > 50%, *P* < 0.1), we performed subgroup analysis according to the nociception measurement, classification of surgery, age, intraoperative opioid, and anesthetic agent used (Table 1).

**Table 1:** Intraoperative opioid administration of subgroup analysis on meta-analysis.

**Subgroup**	**SMD**	***P*-value**	**Effect model**	**Heterogeneity *P*-value**
**Nociception measurement**	
ANI	−0.23 (−0.57, 0.11)	0.19	Random	0.13
NoL	−0.89 (−1.70, −0.08)	0.03	Random	0.001
PPI	−1.97 (−2.91, −1.03)	<0.001	Random	<0.001
SPI	−0.15 (−0.63, 0.32)	0.53	Random	<0.001
**Classification of surgery**
Grade 3 surgery	−0.32 (−0.72, 0.08)	0.11	Random	<0.001
Grade 4 surgery	−1.12 (−2.25, 0.01)	0.05	Random	<0.001
**Age**
≦12 years	−1.43 (−3.03, 0.17)	0.08	Random	<0.001
>12 years	−0.65 (−1.02, −0.29)	<0.001	Random	<0.001
**Intraoperative opioid**	
Fentanyl	−0.71 (−2.03, 0.62)	0.30	Random	<0.001
Remifentanil	−0.59 (−1.08, −0.11)	0.02	Random	<0.001
Sufentinl	−1.14 (−1.96, −0.32)	0.01	Random	<0.001
**Anesthetic agent**	
Intravenous	−0.59 (−1.07, −0.10)	0.02	Random	<0.001
Inhalational	−0.97 (−1.57, −0.37)	0.002	Random	<0.001

SMD, standardized mean difference.

**Figure 8:**
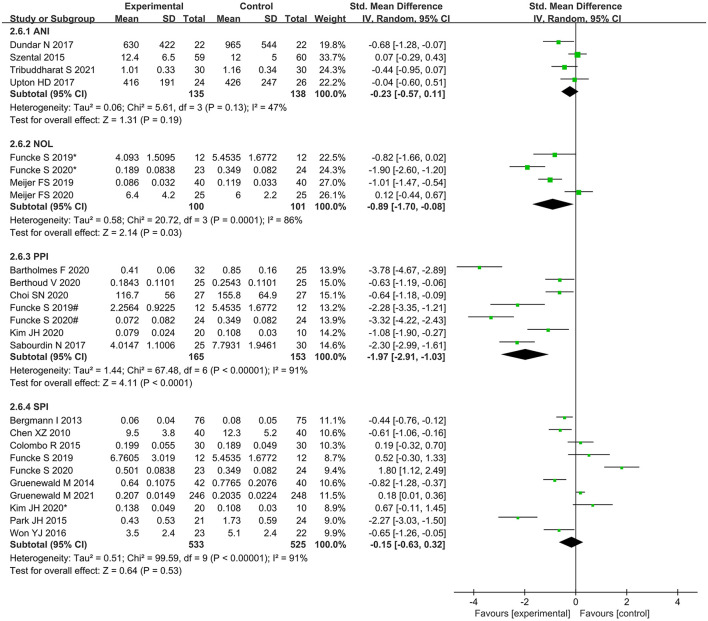
Subgroup analysis according to different nociception monitors.

Intraoperative opioid administration was significantly lower in NoL- and PPI-guided patients than in standard-practice patients; however, no significant differences were found between ANI- and SPI-guided patients and standard-practice patients (Figure 8).

**Figure 9:**
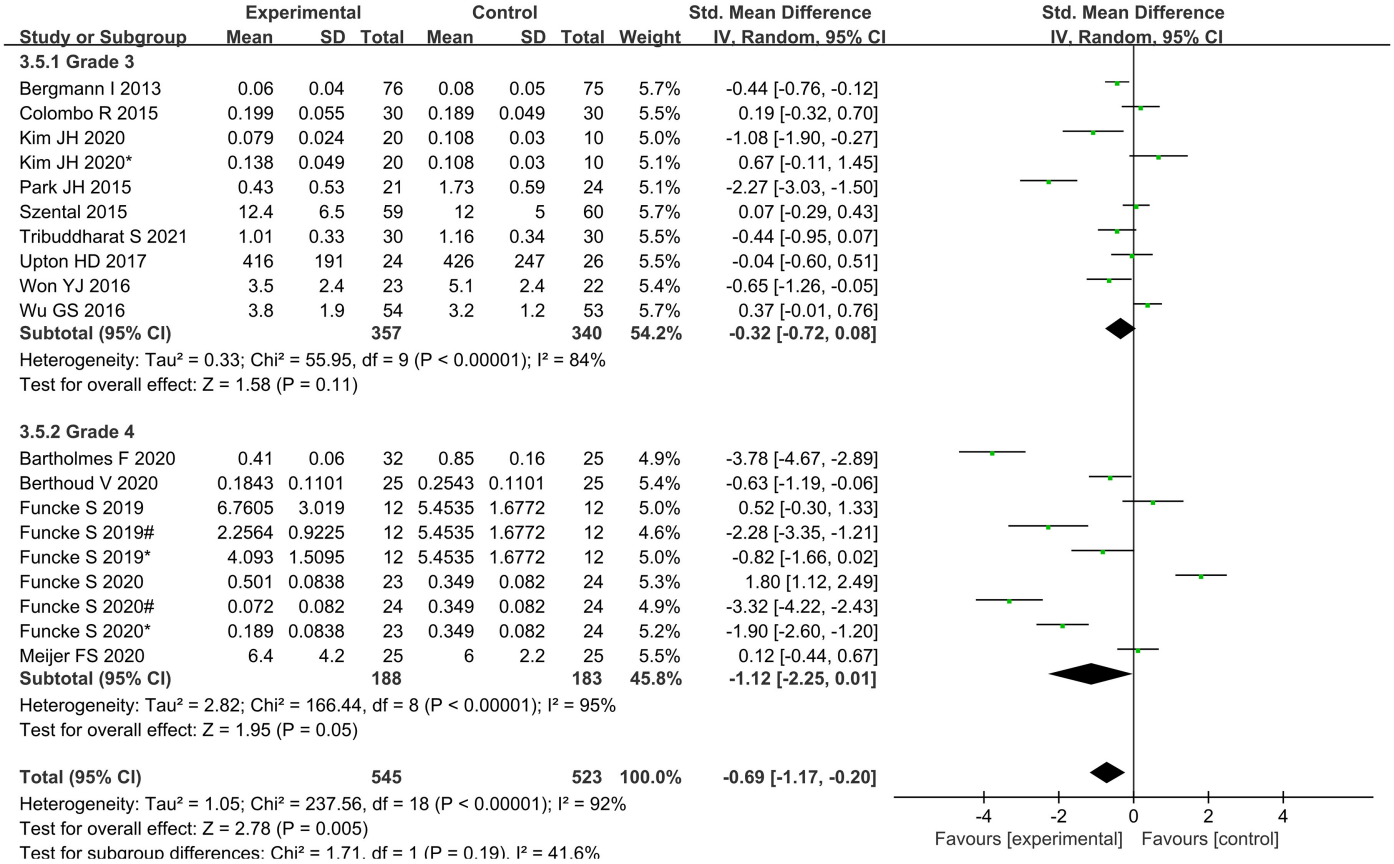
Subgroup analysis according to different classification of surgeries.

Subgroup analysis according to classification of surgery showed that intraoperative opioid administration was significantly lower in Grade 4 surgery, while no significant differences were found in Grade 3 surgery (Figure 9).

**Figure 10:**
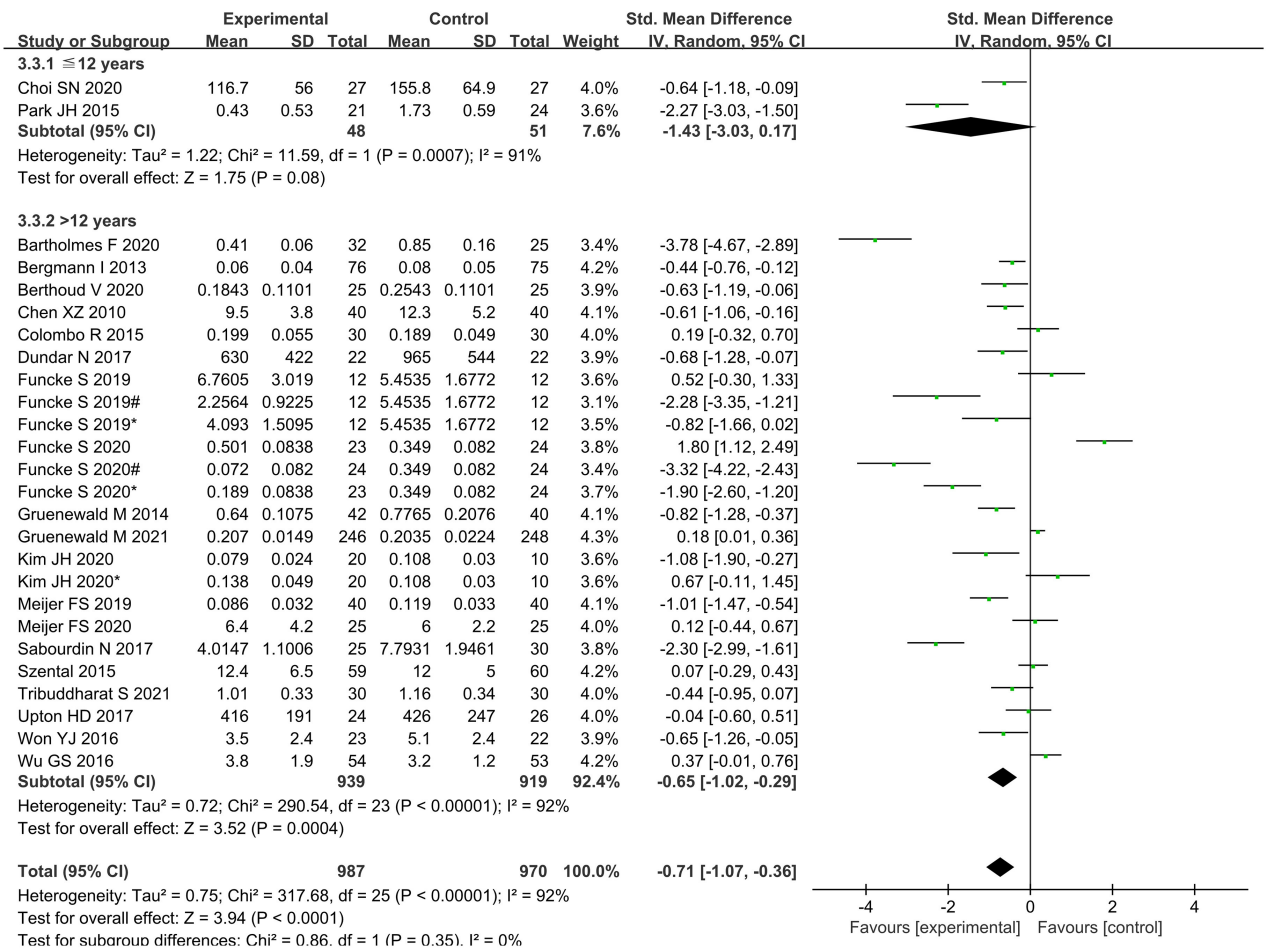
Subgroup analysis according to different ages.

Subgroup analysis according to age showed that intraoperative opioid administration was significantly lower in elder 12 years, while no significant differences were found in younger 12 years (Figure 10).

**Figure 11:**
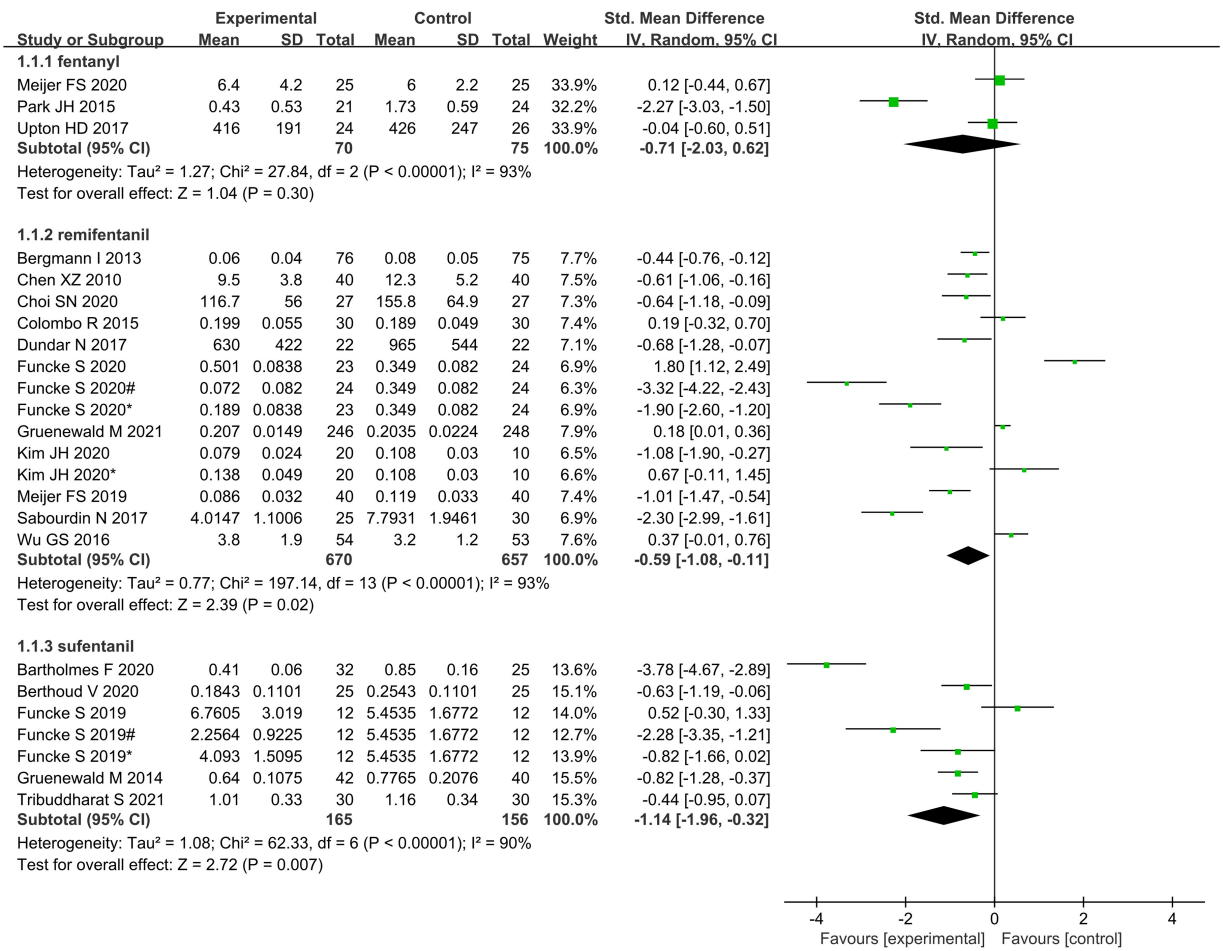
Subgroup analysis according to different kinds of intraoperative opioids.

Subgroup analysis according to type of opioid showed that administration of remifentanil and sufentanil for analgesia was significantly lower in nociception monitor-guided patients than in standard-practice patients. However, administration of fentanyl was not significantly different between the two groups (Figure 11).

**Figure 12:**
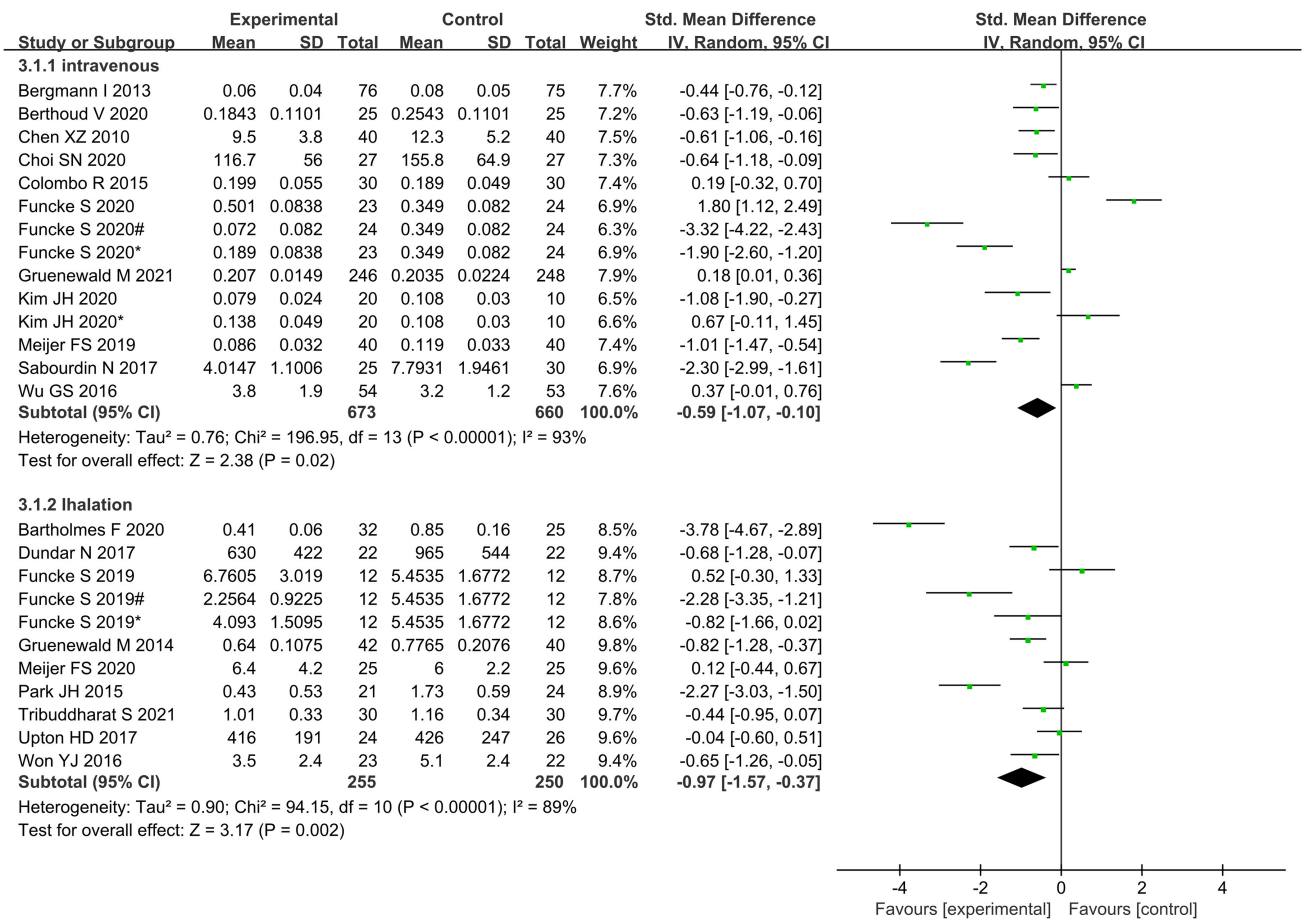
Subgroup analysis according to different anesthesia methods.

Subgroup analysis according to type of anesthetic agent (inhalational vs. intravenous) showed significantly lower opioid administration in nociception monitor-guided patients than in standard-practice patients irrespective of the type of agent used (Figure 12).

The extubation time was significantly shortened in patients who were antagonized by neuromuscular block reversal between monitor-guided group and the standard-practice group. While the extubation time was not shortened in patients without neuromuscular block reversal (Table 2).

**Table 2:** Extubation time of subgroup analysis on meta-analysis.

**Subgroup**	**SMD**	***P*-value**	**Effect model**	**Heterogeneity *P*-value**
Neuromuscular block reversal	−2.43 (−3.59, −1.26)	<0.001	Random	0.740
Non- neuromuscular block reversal	−0.67 (−1.59, 0.25)	0.15	Random	0.004

SMD, standardized mean difference.

### Publication bias

**Figure 13:**
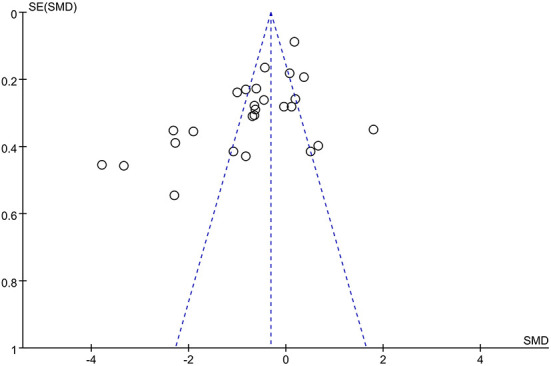
Funnel plot evaluating publication bias.

The funnel plot for all included studies (Figure 13) showed asymmetric distribution around the effect estimate, indicating a slight publication bias in this analysis.

### Sensitivity analysis

Due to the high heterogeneity of primary outcome, sensitivity analysis was performed for intraoperative opioid administration by omitting single study sequentially and recalculating the pooled SMD. The results showed that the overall statistical significance did not change when each single study was omitted (Supplementary Table 4).

## Discussion

This meta-analysis showed that use of nociception monitors can significantly reduce intraoperative opioid administration, without causing increase in postoperative pain or postoperative opioid consumption. Furthermore, nociception monitoring guidance significantly shortened time to extubation and lowered the incidence of PONV.

Nociception monitors are claimed to reflect nociception accurately (30). The primary goal of nociception monitors is to precisely tailor opioid administration according to the individual patient's needs and surgical stimuli. In standard practice, opioids are usually administered as premedication or according to a fixed algorithm during surgery, but this approach could result in excessive intraoperative opioid administration. Our meta-analysis showed significant reduction in intraoperative opioid administration with the use of nociception monitors. Even after the inclusion of evidence from several new studies, our conclusion remains consistent with previous studies (5, 11, 31).

We found that intraoperative opioid administration was significantly lower with NoL or PPI guidance than with standard anesthesia; however, no significant difference was found with ANI or SPI guidance. Jiao et al. (11) found that intraoperative opioid administration was reduced by SPI guidance but not by ANI guidance and suggested that SPI should be preferred over ANI. However, our meta-analysis, which included four recent studies (3, 10, 20, 22) on intraoperative use of SPI, found no advantage with SPI. There could be several explanations for this difference in results. First, prone position during surgery, or laparoscopic surgery with pneumoperitoneum, can increase venous return (32) and thereby affect the SPI values; unfortunately, this information was not available in most of the studies. Second, while SPI values rise rapidly in response to surgical stimuli, they decrease slowly after disappearance of the stimuli. The delayed response of SPI values may cause anesthetists to not reduce the opioid infusion rate. Third, to maintain hemodynamic stability, vasoactive drugs that significantly affect SPI values are inevitably applied (33). Fourth, hypercapnia can increase blood pressure and heart rate (34), and we speculate that carbon dioxide pneumoperitoneum may affect SPI values. Additionally, it should be noted that the reference values for SPI were determined using the data from a large group of adults; the “normal” values of SPI in children may be different. A previous study found that the “ideal” SPI may be significantly influenced by age and is possibly lower in children (35).

Our findings found that in Grade 4 surgery, intraoperative opioid administration was reduced, but this advantage is not evident in Grade 3 surgery. When anesthesiologists perform anesthesia for Grade 4 major surgery, higher doses of opioids under standardized anesthesia may be subjectively used for the several reasons: to inhibit high levels of injury stimulation, to protect the cardiac with opioids (36); to enter the ICU without worrying about delayed awakening. If the nociception monitor is used, these subjective factors can be eliminated and individualized analgesia can be carried out. Therefore, we speculate that nociception monitors have an advantage in time-consuming and difficult surgeries.

Compared with the nociception monitor-guided group and the standard-practice group, the intraoperative opioid administration was significantly lower in the elder 12 years, while there is no differences in younger 12 years. Sabourdin N reported nociception monitors of skin conductance only correlated poorly with conventionally assessed pain levels in children (37). Ledowski T found that a lower SPI target than previously suggested in adults is required to avoid significant postoperative pain (38). Therefore, the reference range of nociception monitors in children needs to be further explored. Moreover there were only two studies in the <12-year-old age group, further studies are needed to confirm this result.

We found that intraoperative remifentanil and sufentanil administration was significantly reduced when nociception monitors were used. Fentanyl consumption was also lower in the nociception monitor-guided group than in the standard-practice group, but the difference was not statistically significant, probably because of the small sample size—only three studies reported use of fentanyl (24, 25, 28).

Intraoperative opioid administration did not vary significantly between the inhalational anesthesia and intravenous anesthesia subgroups. A previous study found that nociception monitor-guided analgesia reduced intraoperative opioid administration during sevoflurane anesthesia but not during propofol anesthesia. A study suggested that this was because opioid requirement is influenced by sedation level (39), and the spinal mechanisms of anesthetic-induced suppression of motor responses differ between sevoflurane and propofol (40). However, it also pointed out that the conclusion may not be reliable as the meta-analysis included only a small number of highly heterogeneous studies. In the present meta-analysis, the majority of the new studies used BIS and entropy indices for monitoring intraoperative sedation, which effectively avoided the effects of excessive sedation on outcomes (11). Our meta-analysis included a relatively larger number of studies, and the result is therefore more reliable. Thus, anesthesia maintenance drugs may not be one of the sources of heterogeneity.

With regard to the secondary endpoints, our findings were consistent with previous studies. Reduced intraoperative opioid administration due to nociception monitors shortens the time to extubation and reduces the incidence of PONV. In addition, we did a subgroup analysis of the extubation time, according to the use of neuromuscular block reversal. The extubation time was significantly shortened in patients who were antagonized by neuromuscular block reversal. While the extubation time was not shortened in patients without neuromuscular block reversal. We speculate that this difference may be due to the fact that the extubation time may be greatly related to the judgment of the anesthesiologist's supervisor when neuromuscular block reversal was not used. Many studies have shown that reasonable control of intraoperative opioid administration could significantly reduce the incidence of opioid-induced hyperalgesia (41). Opioid-induced hyperalgesia affect postoperative pain score and postoperative opioid consumption. Therefore, we speculate that nociception monitor-guided analgesia can reduce the opioid-induced hyperalgesia, thus reducing postoperative pain score and postoperative opioid consumption. Meanwhile, there are many studies suggest that nociception monitors predict postoperative pain (42, 43) and thus decrease the incidence of severe postoperative pain. However, nociception monitor-guided intraoperative opioid titration did not have a significant effect on postoperative pain and postoperative opioid consumption in this meta-analysis. Perhaps due to the surgeries included in the studies are mainly endoscopic surgery, breast surgery and other minimally invasive surgery, the postoperative pain intensity is not severity. In addition, most of the studies we included used multimodal analgesia, such as local anesthetics or Non-Steroidal Anti-Inflammatory drugs (NSAIDs) after surgery, which reduced the incidence of hyperalgesia (44).

A major strength of our study is that this is the largest meta-analysis conducted to date in this field, with a large number of studies and several types of nociception monitors. Moreover, we considered several sources of heterogeneity. However, the study has several limitations. First, although multiple types of nociception monitors were included, some (such as IoC_2_) were used in too few studies. Second, anesthesia protocols for cardiac surgery are different from those for general surgery. Third, the efficacy of nociception monitors may not be same in children and adults. Fourth, some characteristics of the primary research, such as open-label design and receipt of funding from instrument manufacturers, carry a potential risk of bias; however, most of the included studies were published in high-impact journals. Fifth, since some of the original studies did not provide mean and SD, there may still be some bias, although we used median and interquartile spacing for transformation. Last, the sample sizes of the included studies were small; some were only pilot studies.

## Conclusion

In patients undergoing surgery under general anesthesia, nociception monitor-guided analgesia can help reduce intraoperative opioid administration, shorten the extubation time, and lower the incidence of PONV, without causing increase in the degree of postoperative pain and opioid consumption. Increased use of intraoperative nociception monitoring guidance is inevitable. Further large multicenter studies are needed to clarify the role of nociception monitors in pediatric and cardiac anesthesia.

## Data availability statement

The original contributions presented in the study are included in the article/Supplementary material, further inquiries can be directed to the corresponding author.

## Author contributions

DMa and JM participated in the entire procedure including the study design, literature search, data extraction, they also performed the statistical analysis, and revised the manuscript. HC helped to draft the manuscript. DMu, HK, and LY helped to revise the manuscript critically for important intellectual content. All authors read and approved the final manuscript.

## Conflict of interest

The authors declare that the research was conducted in the absence of any commercial or financial relationships that could be construed as a potential conflict of interest.

## Publisher's note

All claims expressed in this article are solely those of the authors and do not necessarily represent those of their affiliated organizations, or those of the publisher, the editors and the reviewers. Any product that may be evaluated in this article, or claim that may be made by its manufacturer, is not guaranteed or endorsed by the publisher.
