# Supplementary material for: Nociception monitors vs. standard practice for titration of opioid administration in general anesthesia: A meta-analysis of randomized controlled trials
Source: Front Med (Lausanne). 2022 Aug 25;9:963185. doi: 10.3389/fmed.2022.963185 (PMC9454957; doi:10.3389/fmed.2022.963185)
Supplement: Supplementary file 3 [file Table_3.DOCX]

| **Supplementary Table 3 \| Summary of study characteristics included in analysis** | | | | | | | | | | | |
| --- | --- | --- | --- | --- | --- | --- | --- | --- | --- | --- | --- |
| Study ID | Journal | Country | Type of surgery | Age | Intervention group | Control group | Guidance of hypnotic effect | Maintenance of anesthesia | Intervention threshold | Opioid | Outcomes measures |
| Bartholmes F 2020 | Dtsch Arztebl Int | Germany | Cardiac surgery | 38-82 | 32 | 25 | BIS | Isoflurane | PPI | Sufentanil | ①②③④⑤ |
| Bergmann I 2013 | Br J Anaesth | Germany | Outpatient orthopaedic surgery | 18-75 | 76 | 75 | SE | Propofol | SPI | Remifentanil | ①②④⑤ |
| Berthoud V 2020 | Sci Rep | France | Cardiac surgery | >18 | 25 | 25 | BIS | Propofol | PPI | Sufentanil | ①②③④⑤ |
| Chen XZ 2010 | Anesthesiology | Germany | Ear–nose–throat surgery | 18-70 | 40 | 40 | BIS | Propofol | SPI | Remifentanil | ①④⑤ |
| Choi SN 2020 | Minerva Anestesiol | Korea | Orthopedic/Urologic/Plastic | 3-12 | 27 | 27 | BIS | Propofol | PPI | Remifentanil | ①②④⑤ |
| Colombo R 2015 | Minerva Anestesiol | Italy | Laparoscopic cholecystectomy | 18-50 | 30 | 30 | SE | Propofol | SPI | Remifentanil | ①④ |
| Dundar N 2017 | J Clin Monit Comput | Turkey | Breast surgery | 18-65 | 22 | 22 | BIS | Sevoflurane | ANI | Remifentanil | ① |
| Funcke S 2019 | Anesth Analg | Germany | Radical retropubic prostatectomy | >18 | 12 | 12 | NA | Sevoflurane | SPI | Sufentanil | ①②③④ |
| Funcke S 2019# | Anesth Analg | Germany | Radical retropubic prostatectomy | >18 | 12 | 12 | NA | Sevoflurane | PPI | Sufentanil | ①②③④ |
| Funcke S 2019* | Anesth Analg | Germany | Radical retropubic prostatectomy | >18 | 12 | 12 | NA | Sevoflurane | NoL | Sufentanil | ①②③④ |
| Funcke S 2020 | Br J Anaesth | Germany | Radical retropubic prostatectomy | >18 | 23 | 24 | NA | Propofol | SPI | Remifentanil | ①②③④⑤ |
| Funcke S 2020# | Br J Anaesth | Germany | Radical retropubic prostatectomy | >18 | 24 | 24 | NA | Propofol | PPI | Remifentanil | ①②③④⑤ |
| Funcke S 2020* | Br J Anaesth | Germany | Radical retropubic prostatectomy | >18 | 23 | 24 | NA | Propofol | NoL | Remifentanil | ①②③④⑤ |
| Gruenewald M 2014 | Br J Anaesth | Germany | Gynaecological / orthopaedic procedures | 18-65 | 42 | 40 | BIS | Sevoflurane | SPI | Sufentanil | ①②③④ |
| Gruenewald M 2021 | Eur J Anaesthesiol | Germany/Finland/ Netherlands/Hungary | Elective surgery demanding general anaesthesia with tracheal intubation | 18-80 | 246 | 248 | SE | Propofol | SPI | Remifentanil | ①④⑤ |
| Kim JH 2020 | Anesth Analg | Korea | Laparoscopic cholecystectomy | 20-65 | 20 | 10 | SE | Propofol | PPI | Remifentanil | ①②④ |
| Kim JH 2020* | Anesth Analg | Korea | Laparoscopic cholecystectomy | 20-65 | 20 | 10 | SE | Propofol | SPI | Remifentanil | ①②④ |
| Meijer FS 2019 | Anesthesiology | Netherlands | Adominal surgical/ urologic/ gynecologic procedures | 18-80 | 40 | 40 | BIS | Propofol | NoL | Remifentanil | ①②③④ |
| Meijer FS 2020 | Br J Anaesth | Netherlands | Laparoscopic/robot-assisted abdominal surgery | >17 | 25 | 25 | BIS | Sevoflurane | NoL | Fentanyl | ①②③④ |
| Park JH 2015 | Anesthesiology | Korea | Adenotonsillectomy | 3-10 | 21 | 24 | SE | Sevoflurane | SPI | Fentanyl | ①②③④⑤ |
| Sabourdin N 2017 | Anesthesiology | France | Gynecologic surgery | 18-60 | 25 | 30 | BIS | Propofol | PPI | Remifentanil | ①②③⑤ |
| Szental 2015 | Br J Anaesth | Australia | Laparoscopic cholecystectomy | 18-75 | 59 | 60 | BIS | Sevoflurane/Desflurane or Propofol | ANI | morphine or fentanyl | ①②③④⑤ |
| Tribuddharat S 2021 | BMC Anesthesiol | Thailand | Mastectomy | 18-75 | 30 | 30 | BIS | Desflurane | ANI | Sufentanil | ①③ |
| Upton HD 2017 | Anesth Analg | Australia | Iumbar discectomy or laminectomy | 18-75 | 24 | 26 | BIS | Sevoflurane | ANI | Fentanyl | ①③⑤ |
| Won YJ 2016 | Medicine | Korea | Thyroidectomy | 20-65 | 23 | 22 | BIS | Sevoflurane | SPI | Oxycodone | ①②③⑤ |
| Wu GS 2016 | Trials | China | Unilateral modified radical mastectomy | 18-65 | 54 | 53 | NA | Propofol | IoC2 | Remifentanil |  |
| # * are from different intervention groups for the same article; BIS: Bispectral index; SE: state entropy; PPI: pupillary pain index; SPI: surgical pleth index; ANI: analgesia nociception index; NoL: nociception level; IoC2: indexes of consciousness NA: no determination in the paper; ①intraoperative opioid administration; ② extubation time; ③:postoperative opioid consumption; ④ pain score; ⑤ postoperative nausea and vomiting | | | | | | | | | | | |
