# Supplementary material for: Nociception monitors vs. standard practice for titration of opioid administration in general anesthesia: A meta-analysis of randomized controlled trials
Source: Front Med (Lausanne). 2022 Aug 25;9:963185. doi: 10.3389/fmed.2022.963185 (PMC9454957; doi:10.3389/fmed.2022.963185)
Supplement: Supplementary file 4 [file Table_4.DOCX]

**Supplementary Table 4 | Sensitivity analysis**

| Removing individual studies | SMD | P | *I*^2^ |
| --- | --- | --- | --- |
| Bartholmes F 2020 | -0.60(-0.93,-0.27) | 0.0004 | 91% |
| Bergmann I 2013 | -0.73(-1.11,-0.35) | 0.0002 | 92% |
| Berthoud V 2020 | -0.72(-1.09,-0.35) | 0.0001 | 92% |
| Chen XZ 2010 | -0.72(-1.09,-0.35) | 0.0001 | 92% |
| Choi SN 2020 | -0.72(-1.09,-0.35) | 0.0001 | 92% |
| Colombo R 2015 | -0.75(-1.12,-0.38) | <0.0001 | 92% |
| Dundar N 2017 | -0.72(-1.08,-0.35) | 0.0001 | 92% |
| Funcke S 2019 | -0.76(-1.12,-0.40) | <0.0001 | 92% |
| Funcke S 2019# | -0.66(-1.02,-0.31) | 0.0003 | 92% |
| Funcke S 2019* | -0.71(-1.07,-0.35) | 0.0001 | 92% |
| Funcke S 2020 | -0.81(-1.15,-0.46) | <0.00001 | 91% |
| Funcke S 2020# | -0.62(-0.95,-0.28) | 0.0004 | 91% |
| Funcke S 2020* | -0.67(-1.02,-0.31) | 0.0002 | 92% |
| Gruenewald M 2014 | -0.71(-1.08,-0.34) | 0.0002 | 92% |
| Gruenewald M 2021 | -0.76(-1.14,-0.38) | 0.0001 | 91% |
| Kim JH 2020 | -0.70(-1.06,-0.34) | 0.0002 | 92% |
| Kim JH 2020* | -0.77(-1.13,-0.40) | <0.0001 | 92% |
| Meijer FS 2019 | -0.70(-1.07,-0.34) | 0.0002 | 92% |
| Meijer FS 2020 | -0.75(-1.12,-0.38) | <0.0001 | 92% |
| Park JH 2015 | -0.65(-1.00,-0.30) | 0.0003 | 92% |
| Sabourdin N 2017 | -0.65(-1.00,-0.30) | 0.0003 | 92% |
| Szental 2015 | -0.75(-1.12,-0.38) | <0.0001 | 92% |
| Tribuddharat S 2021 | -0.73(-1.10,-0.36) | 0.0001 | 92% |
| Upton HD 2017 | -0.74(-1.11,-0.37) | <0.0001 | 92% |
| Won YJ 2016 | -0.72(-1.08,-0.35) | 0.0001 | 92% |
| Wu GS 2016 | -0.76(-1.13,-0.39) | <0.0001 | 92% |

#* are from different intervention groups for the same article; SMD: standardized mean difference
